# Prevalence of Metabolic Syndrome in Chinese Patients With Erythrodermic Psoriasis: A Case-Control Study

**DOI:** 10.3389/fendo.2021.677912

**Published:** 2021-12-14

**Authors:** An-ran Ma, Fang Liu, Runnan Wang, Lanmei Lin, Yilun Wang, Qunyi Li, Xiaonian Lu, Juan Du

**Affiliations:** ^1^ Department of Endocrinology and Metabolism, Huashan Hospital, Fudan University, Shanghai, China; ^2^ Department of Dermatology, Huashan Hospital Affiliated with Fudan University, Shanghai, China; ^3^ Department of Dermatology, Huashan Hospital, Fudan University, Shanghai, China; ^4^ Department of Pharmacy, Huashan Hospital, Fudan University, Shanghai, China

**Keywords:** metabolic syndrome, erythrodermic psoriasis, obesity, hypertension, cardiovascular disease

## Abstract

Erythroderma psoriasis (EP) is a rare and severe form of psoriasis, which is a chronic inflammatory skin disease that usually occurs simultaneously with cardiovascular disease (CVD). Metabolic syndrome (MetS) is a significant precursor of CVD. This study was to investigate the association between EP and MetS in the Chinese population. We conducted a retrospective study on 86 consecutive patients with EP and 100 healthy controls from Huashan Hospital between 2013 and 2018. Demographic, biochemical parameters for MetS, and other relevant data including the severity of EP, family history of EP, age of onset, and treatment history involved in those individuals were recorded. The prevalence of MetS in erythrodermic psoriatic patients was 88.37%, which was significantly higher than that of controls (*P < *0.0001). Erythrodermic psoriatic patients also had a higher prevalence of MetS components, including abdominal obesity, dyslipidemia and hypertension, whereas hyperglycemia was similar. Adjusted for confounding factors, MetS, abdominal obesity, hypertension, smoking and alcohol use were positive independent predictors of EP (odds ratio > 1, *P* < 0.05). The area under the receiver operating characteristic curve calculated from determined risk factors for predicting the EP’s incidence was 0.934 (95% CI 0.902-0.966). There was no correlation between the severity of EP and the prevalence of MetS. Compared with patients with mild EP, patients with moderate-to-severe EP had higher body mass index, waist circumstance and blood pressure (*P* < 0.05). We concluded that the prevalence of MetS and its components was higher in patients with EP. MetS an independent predictor of EP, which can favor CVD and should be encouraged to correct these cardiovascular risk factors aggressively for managing EP.

## Introduction

Psoriasis is a chronic inflammatory skin disease and a significant public health challenge affecting about 2-3% of the population (1). Erythrodermic psoriasis (EP) is a severe form of psoriasis characterized by prominent erythema affecting at least 90% of the body surface with inflammation (2). Owing to extensive and severe skin barrier defects, EP patients can present with systemic complications such as dehydration, infection, edema, cachexia, and electrolyte abnormalities that can be challenging to treat (3, 4). More importantly, EP is also one of the rarest subsets of psoriasis, with an estimated prevalence of 1% to 2.25% among psoriatic patients (3). Therefore, EP treatment is incredibly challenging and usually based on clinical experience and patient co-morbidities because of its low incidence and limited clinical evidence. Accordingly, EP carries with a life-threatening course and an increased risk of mortality compared with other types of psoriasis.

Metabolic syndrome (MetS) is a cluster of conditions involving central obesity, dyslipidemia, hypertension and impaired glucose tolerance and is a strong precursor of cardiovascular diseases, diabetes and incident stroke (5–7). Recently, studies showed that psoriasis was associated with multiple cardiovascular risk factors including obesity, diabetes, dyslipidemia, and MetS (8–11). In addition, cardiovascular death increased in patients with severe psoriasis and has been the most common etiology among them (12). The importance of MetS is that it is associated with a much higher all-cause mortality risk than the individual components (13). To date, the underlying pathogenic mechanisms of EP are not fully elucidated but agents targeting pro-inflammatory cytokines such as tumor necrosis factor α and interleukin 17 inhibitors were effective for EP (14, 15). It was worth noting that a study involving 50 EP patients showed that concurrent illnesses such as hypertension and atherosclerotic heart disease, and excessive alcohol consumption could exacerbate EP, which also suggested the potential association between EP and cardiovascular risk factors (16). Furthermore, the metabolic syndrome was found in 14% of patients with psoriatic erythroderma from a study based on a Tunisian hospital involving 60 EP patients (17). However, most of the available evidence for EP is currently based on small case series and reports, which only describe and summarize the clinical manifestations of EP.

Given the serious comorbidities of the MetS, it should be well-recognized and taken into account in the treatment of individuals with psoriasis. However, there is no data on investigating the association of MetS in patients with EP, probably because it’s rare and severe. The limited clinical evidence available for EP indicates that it necessitates more researches to provide optimal therapeutic options for managing this challenging disease. This study aimed to assess the association between EP and MetS based on the Chinese population, which to our knowledge, is the first case-control study regarding of EP.

## Methods and Materials

### Ethics

The study was conducted according to the guidelines of the Declaration of Helsinki, and approved by the Institutional Review Board of Huashan Hospital, Fudan University (KY2020-1202) and written informed consent was waived for this retrospective analysis.

### Study Population

This case-control study consisted of 86 erythrodermic psoriatic patients (cases) and 100 healthy controls from Huashan Hospital affiliated to Fudan University. In this retrospective study, patients admitted at the hospital’s dermatology outpatient clinic, ward and intensive care unit diagnosed with EP between January 1, 2013 and December 31, 2018 were consecutively enrolled. Patients with EP who had received systemic antipsoriatic treatment including acitretin, methotrexate, oral or intravenous corticosteroids and Tripterygium wilfordii Hook F within three months before admission were excluded in this study. Healthy subjects who attended the hospital’s physical examination center with the exclusion of a history of a diagnosis with generalized scaly, erythematous skin disorders such as EP, psoriasis, pityriasis rosea and eczema were randomly selected simultaneously. EP was defined as a generalized, inflammatory erythematous dermatosis involving at least 90% of the body surface area, with or without associated exfoliation, and having the characteristic clinical and/or histological features of psoriasis with the exclusion of the other main differential diagnoses for erythroderma (2, 18). Subjects with tumors or who have psychological disorders following alcohol abuse were excluded in this study. All subjects from cases and controls were more than 18 years old and were Han Chinese people.

### Methods

We recorded demographic, biochemical and metabolic parameters and other relevant data from all subjects. We collected demographic factors from the cases and the controls, including age, gender, weight, height, and blood pressure on the first day at admission. Body mass index (BMI) was calculated by weight in kilograms divided by the square of height in meters (kg/m^2^).

After fasting overnight (more than 8 hours), venous blood samples were collected in all subjects for analyzing fasting plasma glucose (FPG), serum high-density lipoprotein (HDL)-cholesterol and triglyceride (TG) levels. The serum samples were analyzed at the clinical biochemistry laboratory in Huashan hospital of Fudan University using Roche Cobas 6000 series C501/E601 (Roche Diagnostics GmbH, Mannheim, Germany). FPG was estimated by the hexokinase method, TG and HDL-cholesterol were determined by enzymatic colorimetric assay. We also calculated the prevalence of abdominal obesity, hypertension, impaired glucose tolerance, hypertriglyceridemia and low HDL-cholesterol.

Other relevant data including smoking and drinking status, age of onset for EP, course of psoriasis, family history of psoriasis and the quality of life assessed by using the Dermatology Life Quality Index (DLQI), were also recorded.

The severity of EP was assessed according to a previous study (19), where a moderate-to-severe EP meets at least two of the three following characteristics and a mild EP had less than two characteristics: where a moderate-to-severe EP meets at least two of the three following characteristics and a mild EP had less than two characteristics:

(1) body temperature higher than 37.3°C on admission;
(2) swelling and exudation of more than half of the skin lesion or lower extremity edema;
(3) superficial lymphadenopathy.

### Diagnostic Criteria for MetS

MetS was defined in the presence of three or more modified diagnostic criteria provided by the National Cholesterol Education Program Adult Treatment Panel III (NCEP ATP III) and International Diabetes Federation (IDF) groups for Asians (20):

(1) abdominal obesity (waist circumference ≥90 cm in men and ≥80 cm in women);
(2) reduced HDL cholesterol (HDL cholesterol <1.03 mmol/l in men and <1.30 mmol/l in women);
(3) hypertriglyceridemia (serum triglyceride ≥1.7 mmol/l);
(4) hypertension (systolic blood pressure ≥130 mm Hg and/or diastolic blood pressure ≥85 mm Hg or current use of antihypertensive drugs);
(5) elevated fasting glucose (FPG ≥5.6 mmol/l or having diagnosed diabetes).

### Statistical Analysis

All the statistical analyses were performed using SPSS (version 25.0 SPSS Inc., Chicago, IL, USA). Descriptive statistics were shown as frequency, percentage, and mean ± standard deviation (SD). We adopted univariate analyses to compare the difference between cases and controls by using Student’s t-test for continuous variables, Mann-Whitney U test for non-parametric data, and Fisher’s exact test or Chi-square test for categorical variables. We divided clinical parameters which were continuous variables into two parts according to their medians, and they were allowed to become categorical variables. The relationships between EP and clinical parameters were performed using Spearman correlation analysis. To analyze the independent factors of EP, we performed unconditional logistic regression with backward (Wald) for all indexes with statistical significance (*P* < 0.05) in univariate analysis. Hosmer-Lemeshow test was applied for the goodness of fit for logistic regression models. We used the receiver operating characteristic curve (ROC curve) to determine the best cut-off value of the model by applying Youden’s index. A *P <*0.05 was considered statistically significant.

## Results

### Prevalence of Metabolic Syndrome Among Erythrodermic Psoriatic Patients

In the present case-control study, a total of 186 participants were enrolled, including 86 cases and 100 controls. The demographic and metabolic characteristics of the study population were recorded and shown in **Table 1**. The mean age of the EP patients was 49.57 years (SD = 16.96) and that of the controls was 46.15 years (SD = 15.36). There was a high proportion of male patients in the case group than in the control group (72.09% *vs*. 51.00%, respectively; *P* = 0.003). The proportions of MetS in patients with EP was 88.37%, which was significantly higher than that of the control group (*P <*0.0001).

**Table 1:** Demographic and metabolic features in erythrodermic psoriatic patients (cases) and controls.

	Cases (n=86)	Controls (n=100)	*P* value
Age (years), mean ± SD	49.57 ± 16.96	46.15 ± 15.36	0.151
Sex (male)^†^, n (%)	62 (72.09%)	51 (51.00%)	0.003
BMI^†^, mean ± SD	25.56 ± 2.26	23.62 ± 2.65	<0.0001
Smoker^†^, n (%)	45 (52.33%)	9 (9.00%)	<0.0001
Alcohol use^†^, n (%)	32 (37.21%)	10 (10.00%)	<0.0001
BP^†^ ≥130/85 mmHg, n (%)	85 (98.84%)	50 (50.00%)	<0.0001
FBG ≥5.6 mmol L^-1^, n (%)	40 (46.51%)	34 (34.00%)	0.056
TG^†^ ≥1.7 mmol L^-1^, n (%)	41 (47.67%)	19 (19.00%)	<0.0001
HDL-C^†^ <1.03 mmol L^-1^ (M) or <1.30 mmol L^-1^ (F), n (%)	61 (70.93%)	36 (36.00%)	<0.0001
WC^†^ ≥90 cm (M) or ≥80 cm (F), n (%)	85 (98.84%)	45 (45.00%)	<0.0001
Metabolic syndrome^†^, n (%)	76 (88.37%)	30 (30.00%)	<0.0001

P values refer to the comparison of the cases and controls by independent samples t-test or Mann–Whitney U test for continuous variables, and Fisher’s exact test or chi-square test for categorical variables. P value < 0.05 was considered statistical significance.

^†^, P value < 0.05: There is a significant difference between the case and the control, which has been included in the multiple logistic regression analysis.

BMI, body mass index; BP, blood pressure; FPG, fasting plasma glucose; TG, triglyceride; HDL-C, high-density lipoprotein cholesterol; WC, waist circumference; M, male; F, female.

### Clinical and Biochemical Features in Erythrodermic Psoriatic Patients and Controls

**Figure 1:**
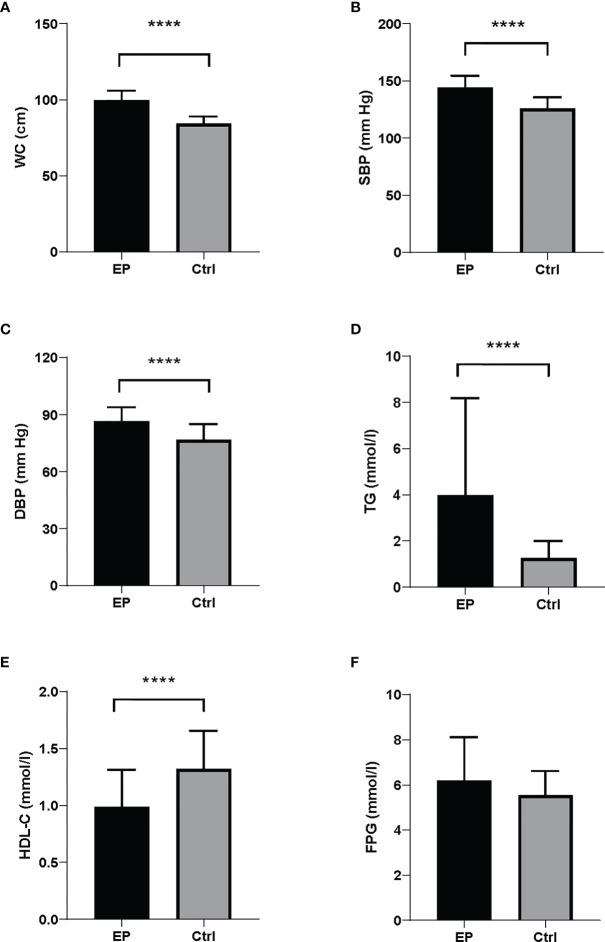
Biochemical parameters for MetS components between EP and controls. There were significant differences in MetS components between the cases and controls. The case group presented higher levels of **(A)** WC, **(B)** SBP, **(C)** DBP and **(D)** TG compared to the control group. **(E)** The case group showed lower levels of HDL-C than controls. **(F)** FPG levels were similar between the cases and controls. Data were shown as mean ± SD. All comparison tests between the two groups were performed using the statistical method of Mann–Whitney U test. ****P < 0.0001. WC, waist circumference; SBP, systolic blood pressure; DBP, diastolic blood pressure; FPG, fasting plasma glucose; HDL-C, high-density lipoprotein cholesterol; TG, triglyceride.

The clinical and biochemical parameters of the study population were presented in **Figure 1** and **Table 1**. Compared with healthy controls, EP patients had a higher BMI (25.56 ± 2.26 *vs*. 23.62 ± 2.65, respectively; *P <*0.0001) and were more frequently smokers (52.33% *vs*. 9.00%, respectively; *P <*0.0001) and drinkers (37.21% *vs*.10.00%, respectively; *P <*0.0001). Moreover, the levels of MetS components were significantly different between the two groups. The levels of waist circumference (*P <*0.0001), TG (*P <*0.0001) and blood pressure (*P <*0.0001) in the case group were markedly higher than that in the control group, and the level of HDL cholesterol (*P <*0.0001) showed the opposite trend, whereas the level of FPG was similar (**Figure 1**). Accordingly, when we converted the continuous variable of biochemical parameters for MetS according to its diagnostic criteria provided by the NCEP ATP III and IDF groups for Asians into a categorical variable, we found the prevalence of abdominal obesity (*P <*0.0001), hypertriglyceridemia (*P <*0.0001) and hypertension (*P <*0.0001) were also higher in the case group, and the prevalence of hyperglycemia was similar between two groups (**Table 1**).

### Correlation of EP With Clinical Characteristics

In order to explore the potential factors that may be associated with the incidence of EP, we converted the continuous variables of clinical parameters into categorical variables according to their medians, and performed the Spearman correlation analysis between EP and clinical characteristics. As indicated in **Table 2**, the levels of blood pressure, waist circumstance, TG, and the prevalence of MetS were positively correlated with the presence of EP levels (r > 0, *P <*0.0001). Besides, male, body weight, BMI, smoking and drinking were also significantly positively correlated with EP levels (r > 0, *P* < 0.05). Height and HDL cholesterol level were negatively related to the levels of EP (r <0, *P* < 0.05). However, age and FPG levels seemed not associated with the levels of EP.

**Table 2:** Correlation analysis between EP and clinical features.

	Cases (n=86)	Controls (n=100)	Spearman Correlation	
			*r*	*P* value
Age ≥48 years, n (%)	49 (56.98%)	47 (47.00%)	0.100	0.176
Sex (male/female)	62 (72.09%)	51 (51.00%)	0.215	0.003
BMI >24.76 kg/m^2^, n (%)	77 (89.53%)	38 (38.00%)	0.236	0.001
Smoker, n (%)	45 (52.33%)	9 (9.00%)	0.462	<0.0001
Alcohol use, n (%)	32 (37.21%)	10 (10.00%)	0.324	<0.0001
BP ≥135 /82 mmHg, n (%)	83 (96.51%)	39 (39.00%)	0.575	<0.0001
FPG ≥5.4 mmol L^-1^, n (%)	40 (46.51%)	44 (44.00%)	0.025	0.733
TG ≥1.19 mmol L^-1^, n (%)	57 (66.28%)	37 (37.00%)	0.292	<0.0001
HDL-C ≥1.04 mmol L^-1^ (M) or ≥1.21 mmol L^-1^ (F), n (%)	24 (27.91%)	71 (71.00%)	-0.430	<0.0001
WC ≥95 cm (M) or ≥86 cm (F), n (%)	81 (94.18%)	13 (13.00%)	0.810	<0.0001
Metabolic syndrome, n (%)	76 (88.37%)	30 (30.00%)	0.588	<0.0001

By converting the continuous variables of clinical parameters into categorical variables according to their medians, data of r and P values were calculated from Spearman correlation analysis. A P value <0.05 was considered as statistically significant.

BMI, body mass index; BP, blood pressure; FPG, fasting plasma glucose; TG, triglyceride; HDL-C, high-density lipoprotein cholesterol; WC, waist circumference.

### Independent Factors That Associated With EP

**Figure 2:**
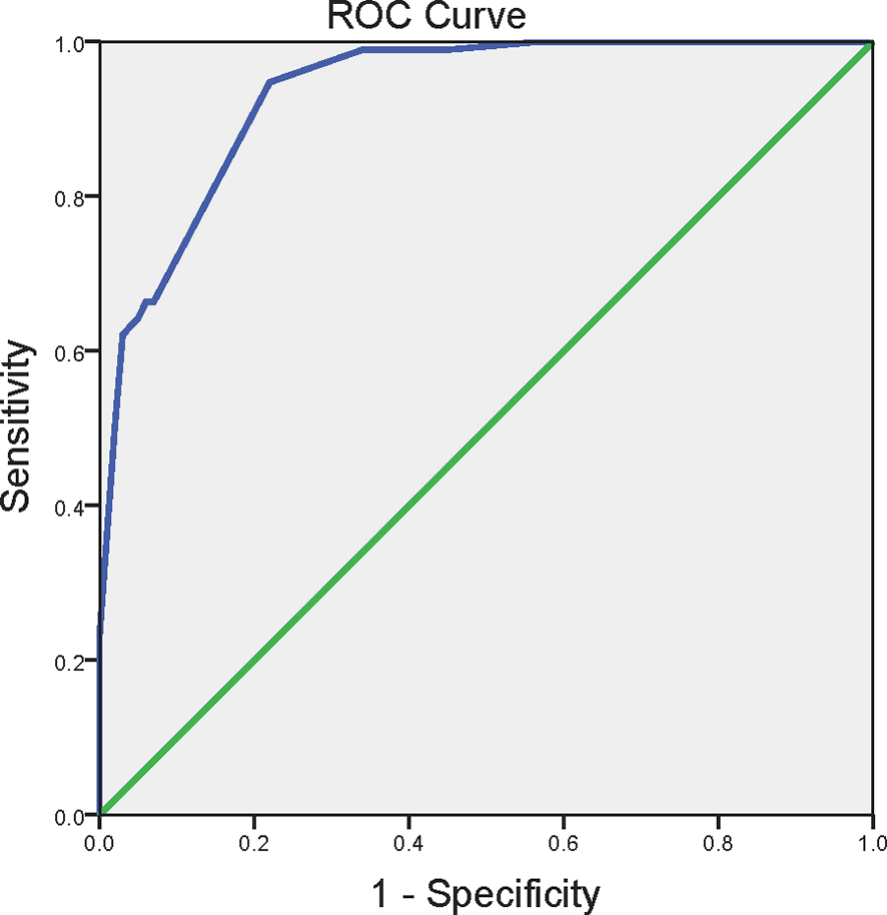
The receiver operating characteristics curve for the prediction of the development of EP. ROC curve for predicting the incidence of EP using the logistic regression function calculated from the presence of abdominal obesity, smoking, alcohol use, hypertension and metabolic syndrome. Abdominal obesity was defined as waist circumference ≥90 cm in men and ≥80 cm in women. Hypertension was defined as systolic blood pressure ≥130 mm Hg and/or diastolic blood pressure ≥85 mm Hg. The metabolic syndrome was defined as in the presence of three or more of the following criteria: abdominal obesity, hypertriglyceridemia, reduced HDL cholesterol, hypertension and elevated fasting glucose. ROC, the receiver operating characteristics curve.

Then, in order to further address the independent factors associated with EP, we conducted the unconditional logistic regression analysis. All indexes with statistical significance (*P* < 0.05) in univariate analysis were incorporated into the regression analysis model. As shown in **Table 3**, MetS independently associated with EP [odds ratio (OR) 4.52, 95% CI 1.54-13.21; *P* = 0.006]. Abdominal obesity [OR 15.60, 95% CI 1.59-153.28; *P* = 0.018], hypertension [OR 30.85, 95% CI 2.09-454.35; *P* = 0.012], smoking [OR 6.20, 95% CI 2.08-18.51; *P* = 0.001] and alcohol use [OR 5.01, 95% CI 1.40-17.93; *P* = 0.013] were also independent predictors of EP, as **Table 3** showed. According to the independent predictors identified by the unconditional logistic regression, we built logit model as follows: Logit (P) = -7.089 + 2.747X_1_ + 3.429X_2_ + 1.825X_3_ + 1.611X_4_ + 1.506X_5_, wherein X_1_ denoted abdominal obesity, X_2_ denoted hypertension, X_3_ denoted smoking, X_4_ denoted alcohol use, X_5_ denoted MetS. To estimate the goodness of fit for logistic regression models, we performed the Hosmer–Lemeshow (HL) test. The HL test results showed that the model is a good fit, with HL statistic=2.873 (*P* = 0.720). Accordingly, the EP risk scores were calculated for the entire study group using this model and the ROC curve was plotted. **Figure 2** showed ROC curves for the prediction of the development of EP using the model. The area under the receiver operating characteristic curve were 0.934 (95% CI 0.902-0.966). According to the ROC curve, the optimal cut-off points of the risk score for predicting incident EP was 0.465 (sensitivity 94.7%, specificity 78.0%).

**Table 3:** Independent factors related to EP by unconditional logistic regression analysis.

Variables	Univariate analysis	Logistic regression	
	*P* value	*P* value	OR (95%CI)
Age	0.151		
Sex	0.003		
BMI	<0.0001		
Smoking^†^	<0.0001	0.001	6.20 (2.08-18.51)
Alcohol use^†^	<0.0001	0.013	5.01 (1.40-17.93)
Hypertension^†^	<0.0001	0.012	30.85 (2.09-454.35)
Hypertriglyceridemia^†^	<0.0001		
Reduced HDL cholesterol^†^	<0.0001		
Abdominal obesity^†^	<0.0001	0.018	15.60 (1.59-153.28)
Metabolic syndrome^†^	<0.0001	0.006	4.52 (1.54-13.21)

^†^present/absent.

All indexes with statistical significance (P < 0.05) in univariate analysis were incorporated into the regression analysis model. OR (95% CI) and corresponding P value were calculated by unconditional logistic regression and P value < 0.05 were considered statistically significant. Abdominal obesity was defined as waist circumference ≥90 cm in men and ≥80 cm in women. Hypertension was defined as systolic blood pressure ≥130 mm Hg and/or diastolic blood pressure ≥85 mm Hg. Reduced HDL cholesterol was defined as HDL cholesterol <1.03 mmol/l in men and <1.30 mmol/l in women. Hypertriglyceridemia was defined as serum triglyceride ≥1.7 mmol/l. The metabolic syndrome was defined as in the presence of three or more of the following criteria: abdominal obesity, hypertriglyceridemia, reduced HDL cholesterol, hypertension and elevated fasting glucose.

OR, odds ratio; CI, confidence interval.

### Metabolic Syndrome and the Severity of EP

We classified the patients as mild or moderate-to-severe EP according to their condition to further investigate whether the prevalence of MetS was correlated to the severity of EP. Comparing EP patients of mild or moderate to severe (**Table 4**), we observed that patients with moderate-to-severe EP had a higher mean age (57.71 ± 14.55 *vs*. 37.71 ± 12.80, *P* < 0.0001), a higher rate of men (82.35% *vs*. 57.14%, *P* = 0.010), an older age of onset (37.71 ± 13.81 *vs*. 25.6 ± 7.77, *P* < 0.0001) and a longer course of psoriasis (19.95 ± 14.24 *vs*. 12.21 ± 10.17, *P* = 0.003). However, there was no difference in the prevalence of MetS in patients with mild or moderate to severe EP (91.43% vs. 90.20%; *P* = 1.000). The waist circumference (*P* = 0.009), systolic blood pressure (*P* = 0.020), body weight (*P* = 0.028) and BMI (*P* = 0.016) were significantly higher in moderate-to-severe EP patients than in mild EP patients. In contrast, we found no significant correlation between psoriasis severity with fasting plasma glucose, triglyceride and HDL cholesterol.

**Table 4:** Descriptive characteristics of subgroups in EP patients.

	Mild EP (n=35)	Moderate-to-severe EP (n=51)	*P* value
Age (years), mean ± SD	37.71 ± 12.80	57.71 ± 14.55	**<0.0001**
Sex (male), n (%)	20 (57.14%)	42 (82.35%)	**0.010**
Height, mean ± SD	169.06 ± 8.13	171.16 ± 6.91	0.216
Weight, mean ± SD	71.40 ± 11.16	76.39 ± 8.40	**0.028**
BMI, mean ± SD	24.86 ± 2.45	26.04 ± 2.01	**0.016**
Smoker, n (%)	14 (40.00%)	31 (60.78%)	0.058
Alcohol use, n (%)	13 (37.14%)	19 (37.25%)	0.992
Age of onset, mean ± SD	25.60 ± 7.77	37.71 ± 13.81	**<0.0001**
Course of psoriasis, mean ± SD	12.21 ± 10.17	19.95 ± 14.24	**0.003**
Family history of psoriasis, n (%)	17 (48.57%)	19 (37.25%)	0.296
DLQI, mean ± SD	29.83 ± 5.91	28.04 ± 6.39	0.192
SBP, mean ± SD	141 ± 10.05	146.35 ± 10.37	**0.020**
DBP, mean ± SD	85.54 ± 8.02	87.16 ± 6.99	0.325
FPG, mean ± SD	5.89 ± 1.73	6.41 ± 2.01	0.224
TG, mean ± SD	4.44 ± 4.57	3.71 ± 3.95	0.441
HDL-C, mean ± SD	0.97 ± 0.27	1.00 ± 0.36	0.730
WC, mean ± SD	97.37 ± 7.25	101.24 ± 5.12	**0.009**
Metabolic syndrome, n (%)	32 (91.43%)	46 (90.20%)	1.000

P values refer to the comparison of the two subgroups by independent samples t-test or Mann–Whitney U test for continuous variables, and Fisher’s exact test or chi-square test for categorical variables. P value < 0.05 was considered statistical significance. Significant differences are in bold.

BMI, body mass index; DLQI, dermatology Life Quality Index; SBP, systolic blood pressure; DBP, diastolic blood pressure; FPG, fasting plasma glucose; TG, triglyceride; HDL-C, high-density lipoprotein cholesterol; WC, waist circumference.

## Discussion

EP is a rare and severe subtype of psoriasis, and the underlying pathogenic mechanisms are not fully elucidated to date. Recently, increasing epidemiologic studies have shown that psoriasis was closely associated with cardiovascular risk factors such as MetS, obesity, hypertension and dyslipidemia (9, 11, 21–23). MetS is a concomitant occurrence of central obesity, dyslipidemia, glucose intolerance, and hypertension, which can favor cardiovascular events and diabetes (5–7). Although many studies found that psoriasis is associated with multiple cardiovascular risk factors including obesity, diabetes, dyslipidemia, and MetS, there is a lack of corresponding data specific to EP. A previous study involving 50 patients with EP showed that 58% of the patients had concurrent cardiovascular illnesses including hypertension, atherosclerotic heart disease, and congestive heart failure (16), suggesting that there may be a close relationship between EP and cardiovascular risk factors.

The prevalence of MetS in our control population was 30.00%, comparable to that recently estimated among 97,098 Chinese adults with 33.9% of the overall population (24). The mean age of 86 erythrodermic psoriatic patients in our study was 49.57 years and 72.09% of the patients were male. This was similar to a previous retrospective epidemiological study involving 60 EP cases, which revealed an approximately 3:1 male-to-female ratio and an average age of 53.7 years (17). The prevalence of smoking in the patients with EP (52.33%) is higher than that in the control group (9.00%), and higher than in the general population in Chinese adults (26.6%) (25). A cross-sectional study consisting of 3953 Shanghai adults through the randomized multistage stratified cluster sampling showed 11.3% of subjects currently drink alcohol (26). This ratio was comparable to that of our control group (10.00%).

Our study demonstrated that the prevalence of MetS was significantly higher in erythrodermic psoriatic patients compared with controls. Patients with moderate-to-severe EP had higher BMI, waist circumference and systolic blood pressure than those with mild EP. We also found that the components of MetS (abdominal obesity hyperglycemia, dyslipidemia, and hypertension) were more significantly common in EP subjects than controls. This was endorsed by some other studies that psoriasis was closely associated with MetS and its components (9, 22, 23). In contrast, Damevska et al. reported no significant difference in the prevalence of the Mets between the untreated plaque patients with psoriasis (24.6%) and the controls (22.9%) (27). There are some several possible explanations for these discrepancy results. Firstly, the patients enrolled in our study were clinically diagnosed with erythrodermic psoriasis, which is a severe form of psoriasis and is different from plaque-type psoriasis in other studies. In addition, differences in nutritional habits, lifestyle, and genetic predisposition among different populations may affect the prevalence of the MetS. The prevalence of MetS in EP patients in our study was 88.37%, higher than the study (24.6%) conducted by Damevska et al.

To further investigate the association between EP and MetS, we established a logistic regression analysis finding that MetS and its components (abdominal obesity and hypertension) were independent risk factors of EP. In addition, we found the other cardiovascular risk factors including smoking and alcohol use, were also independent predictors of EP. These determined risk factors have a good diagnostic performance when predicting the incidence of EP, and we proposed that MetS can be used as a novel risk factor for EP. To date, there are no specific biomarkers to predict or monitor the development of EP, and the use of these observations to find a clinical biomarker for EP may be attempted. It is important to emphasize that the association alone was proven and not causality, although logistic regression analysis provided strong evidence for causality. The underlying mechanism between metabolic comorbidities and EP remains uncertain. There is an urgent need for further prospective research with well-designed longitudinal studies to establish a cause-effect (causal) relationship between EP and MetS.

It is interesting to note that MetS is associated with EP independently of its severity, estimated with body surface area and psoriasis area and severity index scoring (data not shown) and previous classification. Former studies have generally evaluated the condition of patients using the psoriasis area and severity index and body surface area scoring. However, as EP patients were defined as erythematous dermatosis involving at least 90% of the body surface area, psoriasis area and severity index and body surface area scoring may not be appropriate for EP severity evaluation. We adopted the selected characteristics to classify severity because they are common manifestations in EP patients and indicate systemic inflammatory reaction as well as skin barrier destruction (19). This study was cross-sectional, and we cannot determine EP and MetS comes first at the moment. However, the absence of correlation between psoriasis severity and MetS may suggest that obesity favors EP. Obesity, especially abdominal obesity, is now considered as a chronic inflammatory state *via* the continuous release of proinflammatory cytokines, including tumor necrosis factor-α, interleukin 6, which favor MetS and cardiovascular diseases (28). We found a higher BMI level and a higher prevalence of abdominal obesity in EP subjects than those in the controls. In addition, patients with moderate-to-severe EP had high BMI and waist circumference levels compared with mild EP patients. Our results suggested that obesity directly correlated to EP severity. These results were also found in psoriasis patients that a previous epidemiological study identified obesity as a risk factor for psoriasis progression (29). Importantly, agents targeting tumor necrosis factor-α signaling were effective for EP treatment, suggesting that the pathophysiology of EP and obesity shared some cytokines that are known to contribute to features of the MetS, such as hypertension, dyslipidemia, and insulin resistance (30, 31).

The main contribution and novelty of our study were to determine the association between MetS and EP based on 86 Chinese EP patients. To our knowledge, this was the first observational study on the possible association between EP and MetS in China, which fills up the research gap in the field of metabolic syndrome in EP patients. Although EP is considered a subtype of psoriasis, the pathogenic mechanisms involved in EP are not identical to plaque psoriasis and have not been fully elucidated. Studies found that the increased Th2 and Th17 immune response may play a role in EP pathogenesis, endorsed by the higher serum level of interleukin (IL)-13, IL-4, IL-6 and IL-10 in EP patients than patients with plaque psoriasis (32–34). However, plaque psoriasis was considered a Th1-mediated skin disease (35). Conventional drugs for plaque psoriasis, such as oral methotrexate, retinoids, and cyclosporine, are often partially or entirely ineffective in controlling EP (3). Furthermore, the aforementioned conventional drugs showed an unfavorable effect on lipids, hypertension and liver injury and should be used cautiously in psoriatic patients with MetS (36, 37). Since the MetS is one of the positive risk factors of EP based on our study, we proposed that patients with EP should be cautious in using conventional drugs.

However, our study also presented some limitations. Firstly, our study was conducted in a tertiary teaching hospital, where patients enrolled in this study are biased toward having more severe EP. Secondly, it was a cross-sectional study that does not allow the directionality of the association. Thirdly, our results were based on a single-center study conducted among the southeast Chinese population, and the population analyzed may not represent the general population. Fourthly, although our study has included multiple explanatory variables, some variables such as inflammation markers inherent in EP pathogenesis may also result in changes in the response variable (EP). We look forward to enrolling more patients with more explanatory variables to further elucidate the association of MetS and EP in the subsequent prospective study.

In conclusion, we have found the prevalence of MetS and some other cardiovascular risk factors (obesity, dyslipidemia, hypertension, smoking and drinking) were higher in patients with EP, which may play a relevant role in accelerating the morbidity and mortality of the cardiovascular disease. The causality of MetS and EP and the hypothesis that obesity can favor EP needs to be urgently addressed in prospective studies. We suggest that all patients with EP are encouraged to deal with the skin lesions and to correct aggressively their modifiable cardiovascular risk factors, particularly MetS.

## Data Availability Statement

The raw data supporting the conclusions of this article will be made available by the authors, without undue reservation.

## Ethics Statement

The studies involving human participants were reviewed and approved by the Institutional Review Board of Huashan Hospital, Fudan University (KY2020-1202). The ethics committee waived the requirement of written informed consent for participation.

## Author Contributions

JD, XL, and A-rM contributed to the conception and design of the study. JD and XL contributed to collect the clinical data. RW, YW, FL, LL and A-rM analyzed the data. A-rM, JD and QLcontributed to writing manuscript. A-rM, RW and LL gave critical comments to this manuscript and revised the primary manuscript. All authors contributed to the article and approved the submitted version.

## Funding

This research was funded by National Natural Science Foundation of China (NSFC, No. 81673081 and No. 82003382) and Science and Technology Commission of Shanghai Municipality (No. 19ZR1407600).

## Conflict of Interest

The authors declare that the research was conducted in the absence of any commercial or financial relationships that could be construed as a potential conflict of interest.

## Publisher’s Note

All claims expressed in this article are solely those of the authors and do not necessarily represent those of their affiliated organizations, or those of the publisher, the editors and the reviewers. Any product that may be evaluated in this article, or claim that may be made by its manufacturer, is not guaranteed or endorsed by the publisher.
